# From Control to Eradication: The Role of Point-of-Care Testing in Modernising Australia’s Bovine Viral Diarrhoea (BVD) Disease Management

**DOI:** 10.3390/v18060608

**Published:** 2026-05-27

**Authors:** Stephen Ogada, Muhammad Noman Naseem, Shahab Ranjbar, Joshua Aleri, Sheila Cecily Ommeh

**Affiliations:** 1 Centre for Animal Science, Queensland Alliance for Agriculture and Food Innovation, The University of Queensland, Gatton, QLD 4343, Australia; 2 School of Veterinary Science, The University of Queensland, Gatton, QLD 4343, Australia

**Keywords:** bovine viral disease virus, bovine pestivirus, HoBi-like pestivirus, mucosal disease, point-of-care testing (POCT), persistently infected (PI), pen-side diagnostics, surveillance

## Abstract

Bovine Viral Diarrhoea (BVD) is an infectious disease caused by the Bovine Viral Diarrhoea Virus (BVDV), a member of the genus *Orthopestivirus*. The disease remains endemic across Australian beef and dairy production systems, imposing a multi-million-dollar annual burden on animal health, welfare, and industry sustainability. BVDV can be transmitted both horizontally and vertically, with persistently infected (PI) animals serving as the primary source of infection. Rapid identification and subsequent culling of PI animals are fundamental requirements for any successful eradication program. Currently, Australia’s decentralised, non-compulsory approach places the responsibility of biosecurity on individual producers, resulting in a fragmented national landscape. This review proposes that the strategic deployment of rapid, field-deployable point-of-care (POC) diagnostics serves as the transformative catalyst needed for a coordinated national eradication pathway. POC approaches utilising technologies such as lateral flow assays, nucleic acid amplification tests, and biosensors enable real-time, crush-side diagnosis and high-throughput surveillance, proving effective for early detection and control of infectious diseases. When integrated with robust biosecurity measures and optimised vaccination strategies, these POC advancements offer a scientifically sound and commercially viable pathway toward the systematic eradication of BVDV in the Australian cattle industry.

## 1. Introduction

Bovine viral diarrhoea virus (BVDV) is endemic in numerous cattle populations worldwide, including Australia, and is one of the most significant infectious diseases of cattle [1]. BVDV infection in naïve animals and its establishment in a herd can lead to a weakened immune system, severe reproductive losses and increased mortality rates, resulting in significant economic losses [2,3,4]. During gestation, BVDV infection can lead to the emergence of persistently infected animals (PIs). These animals are born with the virus, are immunotolerant to the specific strain present in their tissues, and, when exposed to a cytopathic strain, develop mucosal disease [5,6,7]. Such PIs are destined to produce and shed the virus throughout their lives, representing a significant source of infection for other herd animals. Simulation models suggest that BVDV spreads within a farm, and there is a 60% chance it will become established after a single PI is introduced [1]. Therefore, early and accurate detection of PIs is crucial for successful BVDV control at the herd level.

In Australia, BVDV is endemic, with an approximate national herd-level seroprevalence of 60% that has remained relatively stable since the 1960s, despite variation across production systems, states and territories [1]. Herd-level prevalence shows notable regional variation, with Northern Australian regions, such as Queensland and the Northern Territory, often reporting levels as high as 74.5% in beef cattle [4]. For PIs, one Australian study reported a prevalence of 0.24% among cattle entering feedlots, highlighting the continued circulation of BVDV in the national herd [8]. Overall, more than 80% of surveyed herds, based on regional surveillance and commercial-herd sampling, show some degree of exposure, highlighting widespread pathogen circulation and the need for a comprehensive national eradication initiative [9,10].

Because BVDV pathogenesis is complex and outcomes depend on the timing of infection, accurate diagnosis, effective management, and eventual eradication remain significant challenges in many countries [4,9]. This prompted some countries, such as Sweden, Norway, Denmark, Finland, and Austria, to adopt various strategies to fight the disease. These include voluntary on-farm biosecurity measures and initiatives funded by industry and government, which have led to notable reductions in disease prevalence and economic losses over the long term [11,12]. Despite these successes and the significant economic impact observed in these nations, Australia has yet to establish a national initiative dedicated to BVDV eradication, leaving cattle producers to implement their own biosecurity measures [13].

Herd-level screening is essential for the effective control of BVDV. The ability to collect samples from animals at any location and at any time, and to test them promptly for the virus, provides producers with greater responsiveness. This approach reduces delays associated with transporting samples to a central laboratory and allows for more timely decision-making in managing disease risk. Point-of-care (POC) testing can enable producers to identify PIs and assess herd exposure through flexible sampling. POC testing provides rapid, easy-to-use, and efficient diagnostic solutions for early pathogen detection and intervention [14]. This enables cost-effective strategic prevention, management and control measures that do not necessarily require extensive sample collection and preparation, expensive equipment, or highly skilled personnel. Other benefits include accessibility, improved surveillance and herd monitoring, and on-site convenience.

While vaccination and robust farm biosecurity are vital for controlling BVDV and other infectious diseases, the use of POC testing and ongoing surveillance has become increasingly important for early pathogen detection and disease management [15,16,17,18]. This review examines the potential of POC testing as a strategic approach to support BVDV elimination and eradication efforts in Australia, complementing existing control measures.

## 2. BVDV Management and Control in Australia

### 2.1. BVDV Control Strategies in Australia

BVDV was first reported in Australia during the 1950s and 1960s, but awareness of the disease beyond acute outbreaks remained limited [10,13]. The significant economic impact of BVDV on the Australian beef industry was largely underestimated by producers and veterinarians until the late 1980s [10]. This led to the development of early management and control strategies, which involved sourcing animals from herds with known disease status, testing and quarantining sick or suspect animals, and deliberately exposing cattle to the virus to build immunity. However, in the early 2000s, a licensed inactivated BVDV vaccine called Pestigard^®^ was introduced, showing reasonable effectiveness in Australia [10]. In recent years, economic modelling and estimation have shown that the beef and dairy industries continue to incur significant losses due to BVDV [1,3,19]. Therefore, more systematic and structured strategies have been implemented. These include increased testing to identify PIs and removing them (test-and-cull), as well as improved biosecurity education for producers [1,20]. While maintaining a closed herd system is considered the most effective control strategy for BVDV, it can be challenging, and introducing new animals always carries the risk of inadvertently introducing PIs. Thus, developing an effective vaccination program, implementing testing and culling of PIs, and strengthening biosecurity measures remain central to control efforts [21]. In Australia, such an integrated approach has been recommended, particularly on farms where the disease is active [9].

### 2.2. Challenges Toward BVDV Eradication in Australia

In most cases, the complete eradication of a disease from a country or region requires collaboration and coordinated efforts among multiple partners, including producers, industry, and the government. Many countries have achieved significant reductions in BVDV prevalence and associated losses by implementing voluntary, industry-funded, and/or government-funded programs [11]. This collection of resources, expert skills, and experience from each partner enables the achievement of goals that neither partner could accomplish alone.

The lack of a coordinated national control and eradication program poses a significant obstacle to BVDV eradication in Australia [21,22,23]. This fragmented approach presents additional challenges, notably increased costs associated with comprehensive herd screening on individual farms using traditional laboratory methods [24]. These costs include sample collection and transportation, laboratory testing and analysis, and veterinary consultation. Moreover, there is a risk of reinfection from neighbouring producers who have not implemented control measures, leaving herds that successfully eliminate PIs still vulnerable. The adoption of standardised protocols ensures consistent results across herds, whereas their absence can lead to varied and sometimes ineffective control strategies. National programs facilitate coordinated surveillance, enabling an accurate assessment of the true prevalence and distribution of infectious diseases [9]. Without such programs, understanding the extent of BVDV prevalence in Australian cattle populations and evaluating the success of control and eradication efforts remains difficult. Additionally, national programs promote or enforce the implementation of similar control strategies across herds and farms. This is especially important when individual producers may be reluctant to invest in control measures without confidence that neighbouring producers will adopt comparable strategies.

On-farm biosecurity measures and strategies do not provide a “one-size-fits-all” solution, as demonstrated by Fountain [25] through simulation modelling. However, when integrated with other control measures, including vaccination, these interventions are highly effective and can substantially reduce disease incidence on individual farms while contributing to broader national eradication efforts. Therefore, adopting integrated strategies that combine biosecurity measures, vaccination, and early diagnosis within a coordinated national program represents a crucial approach toward eradicating BVDV in Australia.

## 3. Global BVDV Management, Control and Eradication Status

In many countries, including Australia, the primary rationale for implementing and maintaining control strategies, biosecurity measures and efforts is to improve animal and human health [26]. Over the past 25 years, the control and eradication of BVDV, once considered impossible, have progressed through the implementation of effective systematic control strategies [4,11]. Countries such as Sweden, Norway, Denmark, Finland, and Austria have successfully eradicated BVDV, whereas others, including Ireland, Belgium, Switzerland, and parts of Germany, are close to achieving the same goal [12]. Scandinavian countries initiated their BVDV control programs in the early 1990s and achieved eradication within approximately ten years [11]. Switzerland implemented a coordinated program that did not rely on vaccination and involved direct antigen or viral genome testing of all animals, without prior serological screening. This approach helped reduce the prevalence of PIs to near zero (0.02%) within five years [27]. In Germany, a mandatory nationwide BVDV control program was introduced, combining test-and-cull measures with optional vaccination. This resulted in the culling of 48,000 PIs and a significant decrease in the proportion of cattle herds affected, from 3.44% in 2011 to 0.16% in 2016 [28]. Other notable programs include the Dutch BVDV control program, which has been operational since 2018 and has seen significant success, increasing the proportion of BVDV-free dairy herds from 59% at the start to 89% by the end of 2023 [12]. This control program was initially voluntary but later became mandatory; however, producers remain flexible and can choose an approach that suits their herd. Unlike many other countries that have adopted a single approach, this presents a unique opportunity to assess how different techniques can contribute to achieving BVDV-free herds.

While BVDV control strategies vary across countries, core components, such as herd vaccination and the quick identification and culling of PIs, remain consistent on the path to eradication [21]. These successful national control programs shared several common features, including:

1. *Voluntary and/or nationally coordinated efforts*: Rather than relying on individual voluntary initiatives to manage and control BVDV, producers in these countries, for instance, Scandinavian countries, adopted coordinated and compulsory approaches in their programs [11]. Some of the country programs began as voluntary initiatives; however, they became compulsory as stakeholder and government support grew.
2. *Herd screening and control of BVDV-infected and PIs*: In the Scandinavian model, eradication without vaccination was achieved through a series of procedures involving large-scale bulk-milk serological tests that were conducted to accurately identify herds with active infections and to pre-select farms with an elevated risk of PIs. This was followed by testing individuals, culling detected PIs, and implementing strict biosecurity measures, including regular serological testing and monitoring. Under these conditions, BVDV eradication was achieved within approximately 10 years [5,29].
3. *Surveillance, monitoring and biosecurity measures*: Once PIs are cleared from a herd, close monitoring is conducted regularly to maintain BVDV-free status. This involves routine checks on newborn calves, regular bulk-milk testing, and periodic serological screening of replacement animals. This ongoing surveillance was critical for the early detection of reintroduction events and sustaining long-term freedom from BVDV [5,29].

## 4. The Role of POC Testing in Livestock Disease Control and Eradication

Cattle producers and veterinary professionals worldwide have long relied on several diagnostic methods for disease surveillance, management, and control. These include virus isolation, nucleic acid detection by reverse-transcriptase polymerase chain reaction (RT-PCR), enzyme-linked immunosorbent assay (ELISA) for antigen detection, and immunohistochemistry. However, serological methods, such as the virus neutralisation test and ELISA, are the most widely used for BVDV detection [30].

In Southeast Australia, a survey by McMorrow [31] found that veterinarians primarily used ELISA, agar gel immunodiffusion (AGID), and polymerase chain reaction (PCR) to detect BVDV. Other tests included virus neutralisation, viral isolation, and immunohistochemistry. These tests are among the primary BVDV diagnostic methods used across Australia’s states and territories. While these methods are accurate and widely used, they often require sample collection and transport to centralised laboratories, where tests are performed by skilled personnel and can have long turnaround times. This can result in delays between sampling and diagnosis, especially in remote areas, which may favour the spread of the disease on the farm or at the outbreak site.

The identification of PI animals presents several significant challenges that have hampered BVDV control efforts in Australia. PI cattle with BVDV are serologically negative (antibody-negative) for the homologous strain of the virus they carry due to specific immunotolerance, but they are antigen-positive (virus-positive), and therefore often do not exhibit obvious clinical signs while serving as the primary reservoirs of the virus [6,31]. This fact further emphasises the inherent limitations of serology-based diagnostic methods, such as ELISA, particularly their reduced reliability in identifying PI animals, since their sensitivity is influenced by maternal antibodies in calves younger than 6 months of age [32]. Confirmation of persistent infection in an animal requires multiple tests over several weeks to distinguish it from a transient infection [33]. This increases costs, the risk of misdiagnosis, and the time and logistical complications associated with collecting and transferring samples to testing laboratories. Rapid, efficient, and accurate detection can support successful management, control, and eradication efforts, especially for endemic pathogens and diseases such as BVDV. Point-of-care, on-site, or field-deployable diagnostic tests, when used at the site of a suspected disease outbreak, can enable a faster response that can prevent or mitigate effects on animal and human health.

POC tests are defined as qualitative and quantitative analytical tests performed at the site of patient, host, or sample collection to reduce the time and resources required to obtain diagnostic results [14]. The primary advantage of POC testing is its ability to deliver accurate results quickly, with little to no sample preparation, reduced reliance on sophisticated laboratory infrastructure and specialised personnel, and the ability to be performed on-site or in the field, thereby supporting timely decision-making and action [34,35,36]. The definition of a POC test has continued to evolve as new tools are developed and their applications expand beyond human healthcare institutions. Currently, POC tests are increasingly used in various settings, including veterinary practice and environmental testing, for rapid, on-site diagnosis of animal infectious diseases and for the detection of pathogens in food and environmental samples, such as surface and groundwater [14,37,38]. This has made POC tests increasingly crucial for broader surveillance of animal, human, and environmental health, thereby strengthening the One Health framework.

### 4.1. POC Testing and On-Site Diagnostic Methods

Several POC tests have been developed over the years, incorporating technologies such as immunoassays, molecular diagnostics, microfluidics and biosensors, as well as other novel technologies under development [17]. However, immunoassay-based POC tests are the most common because of their low cost, ease of use, and widespread regulatory acceptance, compared with molecular platforms, which may still require expensive laboratory infrastructure and skilled personnel. Despite these challenges, molecular platforms continue to grow faster than other technologies, owing to their greater analytical specificity, sensitivity, reduced cross-reactivity, ease of modification, and multiplexing potential [17,39,40]. This growth should eventually translate into advanced molecular platforms that can accurately differentiate between acute and persistent BVDV infections faster and at a fraction of the cost. In addition, recent technological advancements have facilitated the development of POC tests across various platforms, such as paper, lab-on-a-chip, portable benchtop instruments, wearable devices (such as ear tags, collars, or nosebands), and mobile phone-assisted tools, which have been used in POC applications [36]. POC devices based on these platforms enable faster, simpler detection of biomolecules across diverse settings and environments, depending on available resources.

#### 4.1.1. Immunoassays

Immunoassays can be developed to target a wide array of analytes, including drugs, small molecules, proteins and peptides, whole pathogens, and their cellular components [41]. The lateral flow assay (LFA) is a type of immunoassay that accounts for the vast majority of POC tests developed and used in clinical and veterinary settings [38]. It typically offers only moderate analytical sensitivity, in the micromolar (μM) range, and variable specificity compared with laboratory-based methods such as ELISA, which reach picomolar to femtomolar (pM–fM) detection limits, and PCR, which often achieves attomolar (aM) detection limits [42]. Because of this substantial difference in sensitivity, conventional LFAs have generally been limited to initial screening applications rather than serving as tools for definitive confirmatory diagnosis. However, recent technological advancements have improved their sensitivity and specificity, bringing them in line with those of centralised laboratories. These include signal amplification using nanomaterials, such as gold nanoparticles [43], and integration with nucleic acid-based techniques, such as recombinase polymerase amplification (RPA-LFA) [42,44]. Notably, many of these technological advancements and strategies are combinatorial, pairing strategies that offer better control over how the fluid moves through the strip, either with an enzyme-driven signal boost or by hooking it up to a reader that detects fluorescence for stronger detection [42].

#### 4.1.2. Molecular Diagnostic Testing

Molecular diagnostic testing is primarily based on nucleic acid amplification and is also known as a nucleic acid amplification test (NAAT). This approach detects and quantifies the DNA or RNA sequences of the pathogen of interest with improved sensitivity and specificity by replicating the target sequences, thereby increasing their concentration and improving detection [41]. Molecular testing methods have continued to evolve and improve over time, incorporating both older technologies and newer ones built on them. These include polymerase chain reaction (PCR), reverse transcription polymerase chain reaction (RT-PCR), quantitative real-time polymerase chain reaction (qPCR), droplet digital polymerase chain reaction (ddPCR), rapid isothermal amplification methods such as Recombinase polymerase amplification (RPA) and LAMP (Loop-mediated Isothermal Amplification), CRISPR-based methods, and metagenomic next-generation sequencing, among others [40].

Initially, nucleic acid amplification required sophisticated, expensive laboratory infrastructure and skilled personnel. The growing demand for more compact, highly accurate benchtop molecular POC testing tools has driven significant progress in miniaturising these instruments without sacrificing reliability. This demand, together with advances in engineering and technology, has prompted the development of more affordable, portable instruments and platforms that can be deployed at the sampling site as either a mobile laboratory (mobile-lab) or a true POC test [40]. Mobile-lab platforms require a controlled workspace and basic laboratory infrastructure but can still be deployed outside central laboratories [45]. However, for these new platforms to be widely used, they need to be affordable, easy to use, and easy to maintain [39]. The reagents used should also require minimal storage conditions and be functional across different environmental conditions.

#### 4.1.3. Microfluidics

Microfluidic devices rely on technologies that process or manipulate small volumes of fluid within channels with micrometre- and nanometre-scale dimensions [46]. These devices are typically small chips, about the size of a palm, that incorporate inlets, microscopic channels, reaction chambers, and sensors for detecting various analytes, such as antibodies and antigens. Micro total analysis systems (μTAS), often called lab-on-a-chip (LOC) systems, and paper-based microfluidic analytical devices (μPAD) are among the most prominent microfluidic technologies [16]. These microfluidic platforms meet the requirements for on-site, accurate, real-time detection and are adaptable for POC testing in veterinary settings because they are rapid, can process multiple samples with low reagent consumption, and are cost-effective [46]. A key strength of microfluidic devices is their ability to handle small sample volumes, which is particularly useful when an adequate sample is not available, especially for infectious diseases [47]. As with immunoassays, recent technologies have enabled the integration of molecular diagnostic methods with microfluidics, thereby improving pathogen detection.

#### 4.1.4. Biosensors

Biosensors are devices that detect the presence of an analyte and its interaction with a specific sensing element, and they include a transducer that converts this interaction into a measurable electronic signal [30]. These devices use various transducers for detection, which can be classified as electrochemical, optical, thermal, and piezoelectric (mass-based) biosensing. Electrochemical biosensors are the most common type and measure electrical changes resulting from the interaction between the analyte and the biological recognition element at the sensor surface [48]. Fluorescence, colourimetric, surface plasmon resonance (SPR), and chemiluminescence-based biosensors are also common and use optical detection methods to measure changes in light properties, such as wavelength, intensity, and colour. In contrast, thermal biosensors measure heat changes during the interaction, whereas piezoelectric or gravimetric biosensors measure mass changes resulting from the interaction between the analyte and sensing molecules, such as quartz crystals on the sensor surface [15]. The type of interaction to be detected has also led to the classification of biosensors in the clinical and veterinary fields by the bio-receptor involved, such as enzymes, antibodies, antigens, nucleic acids, nanobodies, cells, and lipids [48].

Recent advances in quantum computing have also enabled the development of quantum biosensors for detecting infectious diseases [18]. Quantum biosensors take advantage of quantum effects, such as superposition and entanglement, to detect pathogens with greater sensitivity and specificity than traditional approaches, enabling rapid identification even when infectious agents are present at very low levels [49]. Some of these quantum biosensing approaches include quantum plasmonic sensors, quantum dots (QDs), and nitrogen-vacancy (NV) centres [49]. These quantum biosensor devices can be adapted into easy-to-use POC tests. For instance, by integrating quantum dots with specific antibodies, these sensors can target viral biomarkers in animal samples, enabling faster, portable, on-site detection of livestock viruses and diseases, including BVDV.

### 4.2. Currently Available POC and On-Site Tests for Livestock Diseases

Several POC and on-site tests are already used in veterinary practice to detect livestock diseases such as African Swine Fever (ASF), Classical Swine Fever (CSF), Foot-and-mouth disease (FMD) and Brucellosis (Table 1). However, few are recommended by the World Organisation for Animal Health (WOAH). For diseases like ASF, the INgezim^®^ PPA COMPAC is one of the WOAH-recommended commercial blocking ELISAs for detecting African Swine Fever Virus (ASFV) antibodies in pig and wild boar serum and is used to monitor disease freedom alongside other approved diagnostic tests [50]. POC options for detecting ASFV antigens (Ag) or antibodies (Ab) in blood are also commercially available. The INgezim^®^ ASF CROM, a lateral flow assay for detecting ASFV antigens (Ag) or antibodies (Ab) in blood, is known for its fast 15-min turnaround time and enhanced early-detection capabilities compared to older assays [51]. Recently, WOAH recommended a POC test based on lateral flow technology for the diagnosis of Peste des petits ruminants (PPR) by both specialised and non-specialised diagnosticians, making commercial PPRV antigen tests available for field use [52].

Nucleic acid amplification-based methods, such as PCR and qPCR, have been recommended by WOAH as the standard methods for the direct detection of ASFV DNA [53]. This led to the development of a variety of tests, such as the POCKIT^™^ Nucleic Acid Analyzer System [54], which is based on insulated isothermal PCR (iiPCR) technology, and the genesig^™^ q16 platform, which offers portable qPCR [37].

Other notable, highly crucial POC tests that were previously developed include the California mastitis test (CMT), which is easy to use and detects subclinical mastitis in dairy cows. It involves mixing cow’s milk with the test reagent using a paddle and assessing the degree of gelation. The thicker the gel-like substance, the higher the cell count, suggesting an intramammary infection, enabling early detection and treatment [55,56]. Similarly, the Rose Bengal test, one of the earliest modern POC tests, applies the agglutination principle to detect antibodies to Brucella in a patient’s serum. Its high sensitivity, ease of use, speed, and low cost make it valuable for veterinary and human diagnosis to date [57,58].

Building on the success of these established POC assays and platforms, on-site detection of BVDV has been made possible, enabling producers worldwide to determine their herd infection status and progress toward achieving BVDV-free herds. The IDEXX BVDV Ag Point-of-Care Test, a lateral flow test that detects the Erns viral antigen of BVDV in ear-notch tissue samples, is among the primary POC tests used and has demonstrated high accuracy compared with more robust laboratory-based methods, such as qPCR [59]. Additionally, the IDEXX SNAP^®^ BVDV Antigen Test, a rapid, on-site ELISA-based test, has also demonstrated performance comparable to laboratory reference ELISAs conducted in centralised laboratories [60].

### 4.3. POC Testing as a Pathway to BVDV Eradication

**Figure 1:**
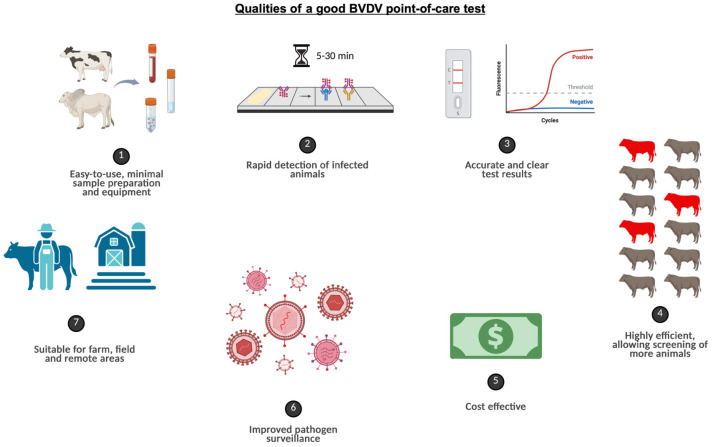
Qualities of a good BVDV POC test. Created in BioRender. Ogada, S. (2026); https://BioRender.com/f2i2j8q (accessed on 20 May 2026).

While centralised laboratory testing remains fundamental to accurate diagnosis and livestock disease management, POC assays and tests can provide valuable insights for controlling endemic pathogens like BVDV. To complement centralised laboratory systems and support BVDV control and eradication initiatives, these POC tests should meet criteria that encourage their adoption in veterinary practice (Figure 1):

- Easy to use, requiring minimal sample preparation and equipment.
- Capable of producing results quickly for the rapid detection of infected animals in a herd.
- Deliver accurate and easy-to-interpret results that correctly identify naïve, transiently infected, and PIs.
- More efficient compared to traditional centralised laboratory methods, thereby enabling the screening of more animals.
- Reduce testing costs to encourage herd screening.
- Offer improved pathogen surveillance, preferably differentiating between variants, strains and lineages.
- Suitable for remote areas where centralised laboratory testing is not easily accessible.

For a POC test to be officially considered for adoption as a standard diagnostic tool, it should ideally meet the criteria set by governing bodies such as the World Health Organization (WHO), the World Organisation for Animal Health (WOAH) and the Food and Agriculture Organization of the United Nations (FAO), even though legislation involving its use still lies with each member country. These well-established and recognised frameworks and criteria for evaluating POC diagnostic tools include the ASSURED (Affordable, Sensitive, Specific, User-friendly, Rapid, and Robust, Equipment-free, and Deliverable to end-users) criteria, developed by the WHO in 2006 [61]. An updated version, REASSURED, was presented by Land [62], which added two additional criteria: R (real-time connectivity) and E (ease of specimen collection and environmental friendliness). Recently, Ochwo [14] proposed an improved framework, FIT-REASSURED, that adds to the ASSURED and REASSURED criteria. This framework assesses the concept of fitness for purpose (FIT) by evaluating the performance suitability of the POC test in question in a use-case scenario and identifying its strengths and weaknesses.

A robust and reliable POC test, when incorporated into routine management at the farm level for testing newborn calves, newly introduced replacements, and sale animals, will support on-farm biosecurity, reduce the risk of persistently infected animals seeding new outbreaks, and add market value to BVDV-tested lines at auction [9,30]. As more farms adopt routine POC screening to identify and cull PI animals, the overall prevalence of BVDV in affected regions tends to decline. This gradual reduction strengthens herd immunity and lowers the overall force of infection in the region, making vaccination programs and stronger biosecurity measures more cost-effective and efficient over the long term [3,63]. With consistent, well-coordinated use of reliable POC tests and adherence to proven industry protocols, consistent, high-quality monitoring data begins to accumulate, laying the foundation for phased national eradication programs. This strategy begins at the farm level, where on-farm choices about individual animals and a single cattle producer’s herd are made. It then progresses to reducing infection rates at the regional scale as more producers adopt the strategy and eventually informs broader national policies. However, this heavily depends on user-friendly, precise POC assays and tests that effectively bridge scientific laboratory insights, veterinary guidance, and routine farm management practices.

## 5. Implementation of POC Testing, Limitations and Future Direction

### 5.1. Implementation of POC Testing in the Australian Context

The Australian cattle production system ranks among the world’s largest and most efficient, particularly in extensive grazing, export volumes, and productivity per animal. As of 30 June 2024, Australia maintained a national cattle herd, beef and dairy combined, of approximately 30.4 million head, including 28.2 million beef cattle [64]. Of these, the state of Queensland, in Northern Australia, holds the largest share, with 13.5 million head. Compared with countries that have successfully eradicated BVDV within their borders, Queensland is significantly larger than Sweden, Norway, Denmark, Finland, and Austria individually, and its cattle herd is larger than all of them combined [64,65]. In northern Australia, beef cattle production typically operates on a more extensive scale across large properties, especially in inland, rangeland-based systems, where vast landscapes support grazing with minimal external inputs [66,67]. In these systems, cattle from different paddocks and properties are commonly mixed during the biannual mustering periods, introducing new cattle into breeding groups at any time of the year [10]. However, in South Australia, beef cattle production differs evidently, aligning with southern Australia’s more temperate, winter-rainfall-dominant systems, which are characterised by intensive production and a different choice of breeds well suited to that climate. Dairy cattle production in Australia also shows regional variation between the northern and subtropical zones and the southern temperate areas. These differences are primarily characterised by distinct calving patterns, herd management strategies, climate-influenced practices, and herd sizes [1]. Such factors significantly influence viral persistence, transmission dynamics, the economic feasibility of control measures, and the likelihood of outbreaks, as discussed by various studies [1,4,10,13,20,25]. These variations in beef and dairy production systems and management practices across different states and territories may entail unique biosecurity requirements, which could pose significant challenges to the design, implementation, and success of a coordinated national effort to control and eradicate BVDV in Australia.

One of the most widely adopted strategies among cattle producers in northern Australia for managing BVDV is to require that any replacement bulls purchased from outside sources be certified free of persistent infection [10]. Seedstock breeders have increasingly made it standard practice to check the PI status of their bulls during routine breeding soundness exams before selling. The widespread adoption of this approach largely stems from the development of reliable antigen-capture ELISAs, which offer high sensitivity and specificity. These tests are straightforward to perform using convenient samples such as hair follicles or ear notches, making it much easier for producers to verify that replacement breeding animals are not PI. Additionally, whole-herd screening is often recommended before the breeding season commences [68]. However, this approach faces technical, temporal, and economic challenges within the framework of the existing centralised laboratory testing system. The collection, transport, and testing of samples, particularly from large herds, are time-consuming. Point-of-care testing (POCT) has the potential to address this significant bottleneck by reducing the time lag and ensuring that results are available, particularly for newly introduced breeding stock. Delays in determining the health status of recently introduced cattle continue to pose significant risks to the main herd, even when quarantine procedures are implemented. This issue is particularly critical in large-scale cattle production systems where mustering is commonly practised. The potential for PI animals to stay undetected in the herd for weeks or even months could significantly impact production.

The economic viability of herd screening for BVDV control and management is also crucial for producers. Herd screening is costly, covering test costs, veterinarian time, and the economic impact of culling PIs. In addition, the economic benefits may be more difficult to quantify in herds with subtle or low clinical signs due to widespread immunity in endemic situations, even if long-term benefits exist [69]. Several cost–benefit studies have demonstrated economic viability for BVDV control, especially prevention-focused approaches, at the herd level, with positive but modest net benefits at the national scale [19,24,70]. Given that BVDV is among the most economically significant endemic diseases in Australian cattle, pursuing a shift toward national elimination could yield greater long-term economic returns, improved animal welfare, and enhanced industry productivity beyond what voluntary herd-level measures alone can achieve. While Australia has the foundational infrastructure needed to move toward the elimination of BVDV, true eradication will depend on securing additional resources, improving coordination, and securing greater financial backing from industry and other stakeholders [4,13].

### 5.2. Limitations and Future Direction

POC diagnostic assays and tests are continually being developed, incorporating advanced technologies such as artificial intelligence to enable timely detection and diagnosis, as well as the efficient implementation of control measures. Although they show promise, they still face key challenges, including limited multiplexing for detecting co-infections, poor interpretability for untrained users, and real-world application hurdles [71]. Immunoassay-based POC tests for detecting BVDV in ear-notch samples have demonstrated very high sensitivity, often approaching or reaching 100% in controlled studies [42]. However, this performance depends on ideal conditions. In real-world field settings, particularly on remote Australian cattle properties, factors such as wide temperature fluctuations, high-dust environments that can lead to sample contamination, and user errors in sample collection and application can reduce the reliability and effectiveness of these tests. Although molecular diagnostic testing platforms offer superior sensitivity and specificity compared with other methods, especially for detecting PIs without symptoms, their implementation as true POC or mobile-lab diagnostics in veterinary medicine remains limited. Many existing options are constrained by instrumentation requirements, nucleic acid extraction steps, specialised training, or high costs, which hinder routine deployment in the field or on farms [40]. Despite recent advances in isothermal nucleic acid amplification and miniaturised NAAT platforms that can be reliably deployed outside centralised laboratories on farms and are suitable for veterinary use, only a limited number of fully automated, affordable, and broadly validated NAAT-based POC tests are currently available. Furthermore, the scarcity of Australian BVDV whole-genome data, which is crucial for designing and developing new molecular diagnostics and vaccines, emphasises the need to address these gaps.

The development of new and improved POC diagnostic assays and tests that can address these challenges, with performance comparable to centralised laboratory-based testing, remains a major research focus in veterinary medicine. These emerging tests are expected to deliver improved analytical sensitivity, greater overall accuracy and repeatability, faster turnaround from sample collection to results, enhanced ability to detect multiple targets simultaneously, reliable and automated interpretation and prediction of results, and scalability across different testing platforms [71]. Moreover, these tests must undergo field assessment through real-world validation under farm, transport, or remote conditions, and then meet standardisation criteria, such as those defined by FIT-REASSURED, to be approved by governments and leading global animal and human health governing bodies [14]. The improved standardisation criterion now enables a full and comprehensive assessment of POC tests, in contrast to the traditional practice, which primarily focused on evaluating diagnostic accuracy using sensitivity and specificity. While these standardisation criteria are useful, fully complying with them is highly challenging.

**Figure 2:**
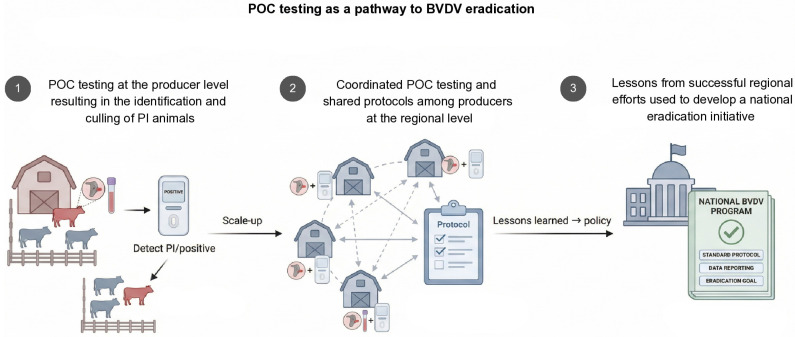
POC testing as a pathway to BVDV eradication. Created in BioRender. Ogada, S. (2026) https://BioRender.com/juzvedo (accessed on 20 May 2026).

Despite progress in developing effective POC tests and platforms, the lack of a coordinated national strategy to employ these platforms and address BVDV prevalence in Australia may still undermine mitigation efforts, as key biosecurity decisions are typically made at the farm level, leading to inconsistent adoption among neighbouring herds. Enhanced coordination among nearby producers, such as through voluntary regional initiatives, shared biosecurity protocols, or collaborative testing and quarantining of introduced stock, could significantly reduce the risk of BVDV transmission across farm boundaries. This is crucial as coordinated biosecurity efforts enable ongoing monitoring and evaluation of implemented BVDV control measures and their outcomes, providing a robust evidence base for timely adjustments and progressive advancement toward national eradication (Figure 2).

**Figure 3:**
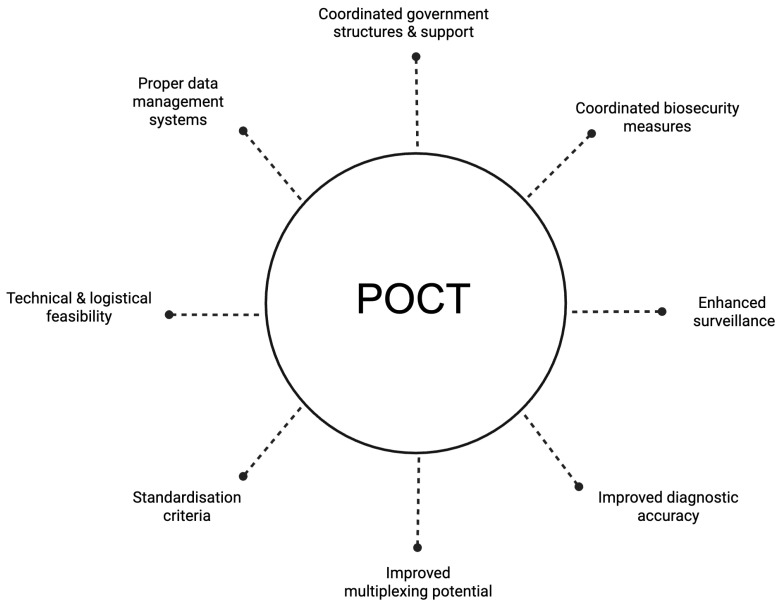
Considerations for POCT integration in a national coordinated program. Created in BioRender. Ogada, S. (2026) https://BioRender.com/5r4y6gc (accessed on 20 May 2026).

While we propose POCT as a crucial component of a coordinated national strategy to control and eradicate BVDV, important issues remain. The technical and logistical feasibility of POCT in field settings may still pose a challenge. Its effective use is contingent on standardised operator training to ensure results are not user-dependent, quality control measures to catch errors, and linkage to confirmatory laboratory diagnosis, particularly for positive or unclear results. The implementation of POCT at the regional level must also be bolstered by coordinated governance structures, such as state authorities, industry bodies, or programs. These structures are essential to facilitate the standardisation of testing protocols, data collection, and reporting procedures across heterogeneous production systems, including feedlots, extensive rangelands, and intensive pasture-based systems. The development of data management systems capable of seamless integration and exchange across platforms, as well as clearly defined standard operating procedures, will also be critical to ensuring consistency, traceability, and scalability towards a coordinated national BVDV eradication program. Finally, effective eradication is closely linked to disease control and animal welfare, requiring the timely and humane management of infected animals, particularly PIs, and strategies to encourage producer compliance and prevent underreporting. Together, these elements enable POCT to function not only as a diagnostic tool but also as an integrated component of a coordinated regional-to-national eradication strategy (Figure 3).

Coordinated biosecurity measures, the development of novel vaccines aligned with specific production systems and environments, innovative therapeutic strategies for PIs, and enhanced surveillance must be integrated into the national eradication strategy. Vaccine research and development, and the implementation of evidence-based immunisation protocols, are essential to address the complex epidemiology of BVDV. Vaccination protocols need to be carefully formulated, guided by empirical evidence, and aligned with the virus’s epidemiological dynamics under field conditions. This should be followed by a rigorous post-vaccination evaluation through serological monitoring to confirm protective efficacy and support the progressive development of herd immunity. Research on BVDV pathogenesis and PI mechanisms can also inform the development of effective therapeutics that specifically target persistently infected animals. This includes elucidating the mechanisms of immune evasion employed by BVDV, such as the actions of viral proteins like Npro, which mediates the degradation of IRF3, and Erns, which suppresses interferon (IFN) production by degrading RNA and concealing pathogen-associated molecular patterns (PAMPs). Collectively, these actions inhibit innate IFN responses and adaptive immunity [7]. Because PIs remain immunotolerant and viremic for life, with no curative treatment currently available, novel antiviral approaches or immunomodulatory interventions are necessary to disrupt viral persistence, reduce shedding, and ultimately support elimination efforts that do not rely solely on detection and culling.

The capacity of other species, such as sheep, to serve as reservoir hosts for the virus emphasises the importance of multi-species surveillance. This is particularly crucial among farmers practising mixed farming or unique management practices, including annual mustering, where cattle roam over vast, sparsely fenced areas, exposing them to other herds, feral animals, and wildlife. In addition, biosecurity monitoring of feral animals, such as feral pigs, wild dogs and deer, should be systematically integrated into the country’s comprehensive BVDV surveillance and control frameworks. These invasive species can also act as potential reservoirs or mechanical vectors, facilitating viral transmission across farms and entire regions. Addressing them through targeted monitoring, population management where feasible, and inclusion in broader wildlife surveillance programs will help ensure a more holistic and effective disease management approach that accounts for Australia’s distinct ecological and agricultural context.

## 6. Conclusions

The long-standing debate over the control and management of BVDV in Australia continues to be driven by extensive discussion, research, and scholarly analysis. As comprehensive cost–benefit analyses continue to yield increasingly compelling evidence, there is a growing national consensus in favour of implementing strategies to control and eventually eradicate BVDV in Australia. To date, the predominant method for managing BVDV infection has centred on identifying and culling PI cattle within herds, a process that relies heavily on reliable diagnostic tests. To improve this strategy, this review suggests that prioritising the enhancement of existing POC tests, developing new ruggedised, high-sensitivity POC platforms, and integrating them with optimised vaccination and biosecurity protocols can help reduce BVDV prevalence and the significant economic burden on Australia’s beef and dairy industries.

This proposed framework advocates for a scalable, bottom-up strategy: beginning with empowered on-farm decision-making, progressing to coordinated regional initiatives, and culminating in a robust national eradication pathway. Ultimately, modernising Australia’s BVDV management not only safeguards the economic and biosecurity future of the domestic beef and dairy sectors but also provides a validated blueprint for disease eradication in large-scale production systems globally. The transition from control to eradication is no longer a question of scientific feasibility but one of strategic implementation.

## Figures and Tables

**Table 1:** Examples of POC and on-site tests developed for the market.

Diagnostic Assay	Method	Analyte	Deployment	Disease
VDRG^®^ FMDV 3Diff/PAN Ag Rapid Kit	Lateral flow	Antigen/Antibody	Point-of-care	FMD
VDRG^®^ ASFV Ag Rapid Kit	Lateral flow	Antigen/Antibody	Point-of-care	ASF
PenCheck^®^	Lateral flow	Antigen/Antibody	Point-of-care	ASF
INgezim^®^ ASF CROM	Lateral flow	Antigen/Antibody	Point-of-care	ASF
IDEXX BVDV Ag Point-of-Care Test	Lateral flow	Antigen/Antibody	Point-of-care	BVDV
INgezim^®^ ASFV/CSFV CROM Ab 25	Duplex lateral flow	Antigen/Antibody	Point-of-care	ASF/CSF
Anigen ASFV Ag Rapid Test	Lateral flow	Antigen/Antibody	Point-of-care	ASF
Rapid NDV Ag	Lateral flow	Antigen/Antibody	Point-of-care	ND
Peste-Test	Lateral flow	Antigen/Antibody	Point-of-care	PPR
POCKIT^™^ Nucleic Acid Analyzer	Nucleic acid amplification	DNA/RNA	Mobile-lab	CSF
genesig^™^ q16 platform	Nucleic acid amplification	DNA/RNA	Mobile-lab	Q fever

FMD: Foot-and-Mouth disease; ASF: African Swine Fever; CSF: Classical Swine fever; PPR: Peste des petits ruminants; ND: Newcastle Disease.

## Data Availability

No new data were created or analysed in this study.

## Abbreviations

The following abbreviations are used in this manuscript:

BVDV	Bovine viral diarrhoea virus
BVD	Bovine viral disease
POC	Point-of-care
POCT	Point-of-care testing
PI	Persistently infected
PIs	Persistently infected animals
